# Lessons Learned from the COVID-19 Vaccination Campaigns in Veneto Region: Population Vaccination Centers as Support for the Traditional Outpatient Model

**DOI:** 10.3390/vaccines11111695

**Published:** 2023-11-07

**Authors:** Sandro Cinquetti, Anna De Polo, Vincenzo Marcotrigiano, Marica Battistin, Erica Bino, Giulia De Mattia, Jacopo Fagherazzi, Nahuel Fiorito, Mattia Manzi, Anna Voltolini, Martina Mognato, Christian Napoli

**Affiliations:** 1 Prevention Department, Local Health Authority “ULSS 1 Dolomiti”, 32100 Belluno, Italy; sandro.cinquetti@aulss1.veneto.it; 2 Hygiene and Public Health Service, Prevention Department, Local Health Authority “ULSS 2 Marca Trevigiana”, 31100 Treviso, Italy; anna.depolo@aulss2.veneto.it; 3 Hygiene and Public Health Service, Prevention Department, Local Health Authority “ULSS 1 Dolomiti”, 32100 Belluno, Italy; marica.battistin@aulss1.veneto.it (M.B.); jacopo.fagherazzi@aulss1.veneto.it (J.F.); nahuel.fiorito@aulss1.veneto.it (N.F.); mattia.manzi@aulss1.veneto.it (M.M.); martina.mognato@aulss1.veneto.it (M.M.); 4 Epidemiology Service, Prevention Department, Local Health Authority “ULSS 1 Dolomiti”, 32100 Belluno, Italy; erica.bino@aulss1.veneto.it; 5 Prevention Department, Provincial Authority for Health Services, “APSS” Autonomous Province of Trento, 38123 Trento, Italy; giulia.demattia@apss.tn.it; 6 Postgraduate Specialization in Hygiene and Preventive Medicine, Department of Cardiac, Thoracic, Vascular Sciences and Public Health, University of Padua, 35122 Padua, Italy; anna.voltolini@studenti.unipd.it; 7 Department of Medical Surgical Sciences and Translational Medicine, Sapienza University of Rome, 00185 Rome, Italy; christian.napoli@uniroma1.it

**Keywords:** organizational model, healthcare workers, vaccination offer, mountain medicine

## Abstract

The extraordinary vaccination campaigns of the COVID-19 pandemic era put organizational and operational systems to the test in numerous territorial contexts. In the Veneto region, the activation of population vaccination centers (CVPs) guaranteed the provision of vaccines to mountain areas. These centers, drive-in and building-based, improved the efficiency of dose administration in relation to similar conditions where healthcare workers (HCWs) were routinely involved in clinics. Overall, a comparison of the two models investigated, with the same numbers of HCWs involved and the same opening hours for the vaccination sites, has shown that the CVPs are able to guarantee three times as many vaccines administered, compared with the traditional outpatient model. This study aims to provide a detailed analysis of the adopted organizational model, highlighting the best practices and improvements required to guarantee a timely and effective public health response, and evaluating the opportunities to deploy these innovative methods actively in a standard context.

## 1. Introduction

Vaccination represents an important challenge from a human, ethical and social point of view, protecting both the individual and the community while helping to reduce the number of individuals susceptible to infection [1,2]. The WHO 2030 Immunization Agenda highlights the importance of offering efficient, universally accessible vaccination services and ensuring the population’s active demand for vaccination. The National Bioethics Committee recommends the creation of vaccination-themed promotion and information campaigns that include effective communications, via media, social networks, and websites, to inform citizens [3,4].

Vaccinations have always been guaranteed for the local health authority ULSS 1 Dolomiti (ULSS 1 Dolomiti), located in the Veneto region in the northeast of Italy, through different operating methods including active calling and free access.

The Resolution of the Veneto Regional Council n. 1935 of 29 November 2016 specifies which actions must be followed to actively offer the vaccinations provided for in the regional vaccination calendar. In particular, the active call involves sending out a vaccination invitation letter, followed by an associated reminder sent via SMS. The identification of users to be invited for vaccination occurs due to the integration of the single regional register with the vaccination register, meaning that information regarding newborn, transferred (immigrants and emigrants), and deceased persons is easily accessible to healthcare workers (HCWs) commonly involved in these work lines [5].

Several studies have shown how the active call for vaccination represents one of the most effective actions by which to improve vaccination coverage [6,7]. In recent years, there has been user engagement and spontaneous access to vaccination (through booking on an online platform or free access) due to multiple information communication campaigns via brochures and social campaigns. To recover those vaccinations that saw a drop in coverage during the COVID-19 pandemic, catch-up interventions have also been put in place [8]. In the pre-COVID-19 era, the vaccinations envisaged by the National Vaccine Prevention Plan (PNPV), aimed at children, adolescents and adults, were primarily offered within the clinics located in the local area in order to guarantee coverage in the most peripheral areas and, consequently, to maximize accessibility to the population.

The emergency situation caused by the COVID-19 pandemic has led to the need to rapidly vaccinate as many people as possible against the disease, with the aim of increasing vaccination coverage in the population. The usual vaccination clinics were not sufficient to facilitate the timely achievement of this objective. With Resolution no. 239 of the Veneto Regional Council, issued on the 2nd of March 2021, the Veneto region has released a manual regarding the organization of population vaccination centers (CVP) in order to guarantee rapid mass immunization, while still maintaining optimal levels of efficiency and safety. Each CVP is characterized by an entry, a moment of personal identification of the user, an evaluation of the user’s medical history to verify their suitability for vaccination, the administration of the vaccine upon registration, a post-vaccination wait, and an exit from the CVP [9].

Another rapid, effective, and safe strategy that allows mass vaccination is represented by the “drive-in”: this mode, widely used for performing COVID swabs, requires the user to receive the vaccination while remaining aboard their car. For its implementation, large spaces are necessary to ensure post-vaccination rest. This technique had already been tested in the summer of 2020 in ULSS 1 Dolomiti for the delivery of anti-TBE (tick-borne encephalitis or tick bite encephalitis) and anti-pneumococcal vaccinations to subjects over 65 years of age [10]. The drive-in facilities were then used in the post-COVID-19 period for the organization of vaccination campaigns, including TBE and pneumococcus, and for the recovery of vaccinations in adolescents (e.g., HPV, menACWY). These new operating methods require a substantial commitment of economic resources and personnel, but they allow a large number of people to be vaccinated within a short time frame.

This study aims to provide a detailed critical analysis of the vaccination operational models implemented during and after the COVID-19 pandemic in the Veneto region, highlighting the best practices in the organization of the provision of vaccinations to the population. These practices, from an emergency context, may not only be passed down for possible future emergency scenarios, but also transfer well to ordinary practice.

## 2. Materials and Methods

### 2.1. Background

The ULSS 1 Dolomiti includes the predominantly mountainous territory of the province of Belluno (3.678 km^2^), being the largest province of Veneto. The provincial population density is 264.6 inhabitants/km^2^ for a total of 197.751 inhabitants, marking a decrease of almost 2% from 2017 to 2022.

Population aging is one of the most important aspects of demographics: since 2018, the average age of the population has increased from 47.3 to 48.5 years and is approximately two years higher than the regional average, while the old age index (percentage ratio between the number of people aged over 65 and the number of young people up to 14 years old) went from 222.8 to 254. The population aging trend will continue in the coming years, with an aging index of 303.8 forecast for 2030 [11].

### 2.2. Traditional Outpatient Vaccination Model Session Management

‘Vaccination session’ is understood as the temporal and operational path that begins with the opening of the vaccination clinic and ends with its closure. It is divided into different phases, including planning, communication, administration, registration, and organization of physical spaces and logistics, in order to guarantee the safety of the users and the HCWs in terms of the quality and appropriateness of the services provided [12,13].

In the traditional model, the administration of vaccines takes place in the dedicated clinics of the Hygiene and Public Health Service (SISP) belonging to the Prevention Department, according to a process logic comprising the following phases:

- Preparation of the vaccination session—the HCW prepares the physical environment for the session, checks the fridge’s temperature, inspects the batches and expiry dates of the vaccines to be used, and prepares the necessary and emergency material and access to a dedicated computer software;
- Reception—users present themselves at the dedicated clinic and the HCW welcomes them, identifies them and, in the case of a minor, verifies that the parents have received the relevant information, that they have been adequately informed by their pediatrician, and are in possession of previous vaccination documentation;
- Counseling—the HCW checks the user’s vaccination status, explains the risks and benefits of the specific vaccination, responds to requests for clarification and takes the opportunity to propose the other vaccinations recommended for age and pathology;
- Filling of the medical history—the HCW compiles the medical history and, if the latter is negative, proceeds autonomously with vaccination administration. In the event that precautions are required or contraindications emerge, the HCW contacts the physician responsible for the session, present in the facility, for the final decision (vaccination, postponement, or exemption);
- Administration and registration—the HCW checks the vaccination to be performed, checks the batch, the expiry date, the conformity of the preparation and, once consent has been acquired, proceeds with administration. In this phase, information is provided about the most common side effects, illustrating the methods and timing used to report the events. Once this phase is completed, a registration of the vaccination is performed using a dedicated IT platform;
- Observation—the HCW suggests that users stay in the waiting room for at least 15 min so that clinicians can intervene immediately should rapid onset adverse reaction occur.

Using this method of organization, the SISP staff required for the optimal management of the vaccination session normally include the following:

- One physician, responsible for the session and present in the facility;
- Two health visitors, or other dedicated staff, per clinic or vaccination cubicles.

Users are called at a different frequency depending on whether the planned vaccinations are pediatric, for the adult population, or provided as part of dedicated vaccination campaigns. Regarding pediatric vaccinations, invitations are scheduled with a frequency that includes one child every 10–15 min in order to allow HCWs to inform and support parents, especially for the first vaccinations; for adults, an invitation is expected every 5–10 min, which varies based on the type of session and vaccine administered.

Each clinic is generally organized into shifts with the following division:

- 4 h morning shifts (from 8 a.m. to 12 a.m.);
- 2.5 h afternoon shifts (from 2 p.m. to 4.30 p.m.);

For a total of 6.5 h in total, treating a maximum of 39 children or 78 adults in one day.

### 2.3. New Models Adopted during the Anti-SARS-CoV-2 Vaccination Campaign

In the pre-pandemic period, 16 local vaccination points and 5 hospital facilities were active in ULSS 1 Dolomiti, the latter being typically active for tetanus vaccinations carried out in emergency scenarios by emergency units and for flu vaccinations for the local health authority’s workers in winter season. The placement of the CVPs was based on the ease of availability of supplies, the catchment area, the territorial logistics, the technical and IT assistance and cleaning facilities. In the feasibility assessment of CVPs, each with its own different features, the healthcare, administrative and technical staff of the local health authority were involved, supported by the prevention department, the hospital medical management, the emergency–urgency department, the prevention and protection service, the supply and logistics service and the technical service. In summer 2020, with a large demand for vaccinations coinciding with the start of the free vaccination campaign against TBE, the ULSS 1 Dolomiti adopted an innovative drive-in vaccination method that, by 2021, proved to be the method of choice for the mountainous area of the province of Belluno. Used alongside some CVP of the traditional “building” type (i.e., in large pre-existing buildings reconverted ad hoc), this method was able to make up for the unprecedented vaccination demand resulting from the anti-SARS-CoV-2 vaccination campaign [14].

**Figure 1:**
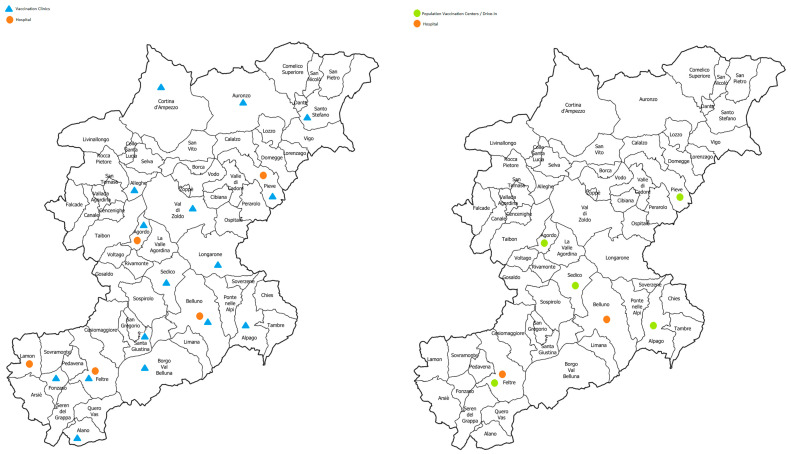
Distribution of vaccination access points in the ULSS 1 Dolomiti territory in 2019 (on the left—the orange dots represent the hospital facilities and the green triangles represent the local clinics belonging to the prevention department) and in 2021 (on the right—the orange dots represent hospital facilities and the green dots represent drive-in and “building” CVPs belonging to the prevention department).

Figure 1 shows the distribution of vaccination points in 2019 and 2021.

These structures were located outside of the hospital perimeter so as not to interfere with internal traffic. The pre-existing vaccination clinics at the prevention department in the central and peripheral sites, on the other hand, did not allow access to the high proportion of the population who needed it in the extraordinary circumstances of the anti-SARS-CoV-2 vaccination campaign, both due to insufficient space and specific HCW enlistment.

In fact, the pandemic led to a need to quickly and extensively provide vaccination to all citizens: the first solution, in a mountainous area like that of the ULSS 1 Dolomiti, was the drive-in method, already in use to perform swabs, and subsequently the creation of buildings largely dedicated to CVPs.

Systems were therefore assessed based on their capacity to accommodate users, the ease of access or use, and the possibility of channeling cars without impeding public streets. Having to cope with the harsh climate typical of the province of Belluno, the CVPs, both drive-in and building-based, were equipped with heating solutions, as well as tensile structures suitable for adverse weather and climatic conditions.

The vaccination campaign was structured based on age group: elderly and frail people were involved at the beginning and, having by nature potential difficulties in reaching the facilities, the drive-in method reduced travel in a significant and welcome manner for this group. Once the first emergency phase was over, due to the progressive involvement of ever lower age groups and the ever wider availability of vaccination supplies, the most effective solution was the identification of large buildings with wide access. The primary features of this are detailed below:

- Personnel involved: The preparation of the vaccine doses, the training of the session staff and the distribution of the vaccines was performed with the involvement of hospital pharmacy and civil protection volunteers. The management of the CVP was guaranteed by the prevention department staff, who were actively involved in both the organizational and administration phases. The civil protection department and voluntary associations guaranteed that the reception and post-vaccination control activities would take place in an easy manner. In consideration of the large number of users to be vaccinated in the shortest possible time, specific objective projects were begun in order to allow users to benefit from the support of hospital personnel normally involved in different lines of work, while at the same time expanding the vaccination lines and the types on offer. Employment contracts were negotiated with HCWs (especially health visitors, nurses, and environmental health officers), including those who were retired and available, in order to support the SISP’s activities. To guarantee data entry and the registration of the vaccinations, a contract was activated with an external cooperative to guarantee the presence of dedicated administrative staff to support the HCWs. Some private users also offered their support to the ULSS 1 Dolomiti during the anti-SARS-CoV-2 vaccination campaign: this was the case with a large eyewear industry in the Belluno area, which provided its own trained nurses and administrative staff for the occasion; furthermore, a private health center provided a supply of its internal healthcare staff. Additionally, for the management of any emergencies, the emergency department operational units of the hospitals located near the CVPs were involved in the sessions planning. The exception to this was drive-in CVPs, which were supervised by an ambulance crew located on site.
- CVP drive-in: The drive-ins were equipped with a tensile structure containing computer stations and medical supplies. Containers were placed inside the main tensile structure for vaccine preparation, for the storage of medical materials (aided by a refrigerator for storing vaccines), and for first aid. Each drive-in was equipped with an independent electricity network, internet connection, toilets for staff and users, and road signs, both along the access road and on site. Volunteers directed users who accessed the site in their own vehicle along the route to be followed in order to complete the vaccination process. While patients were waiting in the queue, the HCWs filled in the medical history and the administrative staff registered the user in the dedicated software with the support of data taken from health insurance cards (HIC). Once inside the tensile structure, the vaccination staff reviewed the certified medical history and proceeded to conduct the vaccination. Post-vaccination surveillance was carried out in delimited areas adjacent to the healthcare area, constantly monitored by support staff.
- Traditional or building CVP: Building CVPs were each equipped with a large room divided into a pre-vaccination area, a vaccination area, and a post-vaccination surveillance area. There was also a dilution area equipped with fridges and a first aid area that possessed an emergency trolley. Signs were placed along the access road and inside the building. Users accessed the center and were welcomed by volunteer staff who performed an initial recognition using the HIC, checked the appointment and provided the medical history to be filled out. The user was then directed to the waiting room in order to fill out the medical history form; subsequently, forms were sorted into the cubicles. In fact, vaccination cubicles were set up in the CVPs to allow for the medical history recovery and the vaccination to be carried out, guaranteeing the user the necessary privacy. Inside the cubicle, there was a table with a laptop and printer, which was useful for checking the vaccination situation, viewing the medical history and recording. CVPs also provided a seat for the user and a trolley for the material useful for the vaccination procedure, with waste containers provided to allow for separate waste collection. Inside the cubicle, the user was assisted by HCWs who checked patient medical history and proceeded to administer the vaccination. The process was aided by additional administrative staff who were responsible for registering the vaccination at the dedicated portal and delivering a vaccination certificate. In the event that administrative staff were outside the cubicle, the user was registered before or after the vaccination in the dedicated workstations. At the end of the vaccination, the user was seated in the section of the room dedicated to post-vaccination surveillance, before being dismissed.

At the ULSS 1 Dolomiti, there are currently 11 vaccination clinics. These were reopened in 2023 after the interruptions due to the COVID-19 pandemic. Table 1 shows how they are distributed throughout the whole area to make it easier for users to access the services, while Table 2 shows their operationalization of strengths and areas for improvement.

### 2.4. Recovery Campaigns

Once the first phase of the anti-SARS-CoV-2 vaccination campaign was concluded, with the need to process the waiting lists created due to the suspension of ordinary vaccinations during the lockdown period, the prevention department used the spaces and resources allocated to anti-SARS-CoV-2 vaccinations to recover pending routine vaccinations in the shortest possible time. CVPs were thus used for the anti-TBE vaccination and for the anti-HPV, anti-pneumococcal, anti-herpes zoster vaccination campaigns and for boosters intended for 14-year-olds.

In the case of vaccinations for patients assessed as being at a high risk of adverse reactions, users were booked at a dedicated clinic located inside the Belluno Hub Hospital. In our local health authority, as of 30 June 2023, 23,156 anti-SARS-CoV-2 vaccinations have been administered to vulnerable or extremely vulnerable users, of which 1241 were administered in a controlled and protected healthcare environment. During the entire vaccination campaign, 460 adverse drug reactions (ADRs) were notified to the prevention department and recorded, none of which resulted in serious acute effects requiring hospitalization. In light of these data, the ADRs recorded in the entire anti-SARS-CoV-2 vaccination campaign (463,186 doses) are 0.1%.

## 3. Results

In summer 2020, due to the large demand for vaccinations resulting from the start of the free vaccination campaign against TBE, the ULSS 1 Dolomiti adopted the innovative drive-in vaccination method. This was initially only performed in the external area of the Belluno Hub Hospital. The adoption of this method allowed 12,152 doses of anti-TBE vaccine to be administered in the summer period (24 June–31 August 2020). Since this model made it possible to vaccinate a large number of users, while at the same time minimizing the risks of contagion linked to the SARS-CoV-2 pandemic, the local health authority, in light of the territory extension and the difficulties in connecting all the provincial areas, activated other drive-ins located throughout the area (Cadore, Agordo, Belluno and Feltre). These centers allowed for an initial recovery of adolescent vaccinations (anti-papilloma virus, fifth doses DTPa-IPV and meningococcal ACWY), suspended due to the pandemic, and to manage the anti-flu and anti-pneumococcal campaign in the autumn months, convening the active population of 60–65-year-olds. Furthermore, the anti-SARS-CoV-2 vaccination campaign began in the aforementioned drive-ins, which saw the administration of 344 doses of the vaccine in the last days of 2020. Overall, from the start of the drive-ins, until the end of 2020, 27,988 vaccine doses were administered, most of which were given to the adult population (catch-up of 14-year-olds also fall within this range) and a smaller part to the pediatric population (2724 administrations, almost exclusively anti-TBE). In 2021, in consideration of the evolution of the anti-SARS-CoV-2 vaccination campaign and in order to expand the offer, new centers were activated: the temporary drive-in in Cortina D’Ampezzo at the ice rink, the drive-in in Paludi (readapted by converting the previous COVID swab point) and the CVP building Palaskating Sedico. Thanks to these new centers, it was possible to administer over 300,000 doses of the anti-SARS-CoV-2 vaccine over the course of the entire year. Based on the efficiency and safety demonstrated in the previous year, anti-TBE vaccination sessions were also organized during 2021 at the traditional and drive-in CVPs (6089 doses administered) and the vaccinations covered by the vaccination calendar, which involved both 5–6-year-olds and teenagers. With reference to the anti-SARS-CoV-2 vaccination campaign, on 13 March 2021, an open-day for people aged over 80, the cohort with the highest number of vaccinations, was recorded at the ULSS 1 Dolomiti: 2028 vaccines were administered at the 4 drive-ins located in the province. To guarantee this activity, 40 HCWs were employed (18 medical doctors, 22 health visitors and nurses) with the support of local volunteers and law enforcement in order to manage the reception and waiting phases. By adding the opening hours of the four supply points, the result was 34 h of activity. This is equal to 2040 min, which is equivalent to a rate of one vaccination per minute. Comparing these data with the estimate of the maximum capacity achievable in the outpatient context of the ULSS 1 Dolomiti, which saw 16 clinics, each active for 7 h of activity (112 h of total activity), it is clear that, in order to guarantee the simultaneous functioning of the vaccination points, it would have been necessary to employ 35 HCWs (16 medical doctors, 19 health visitors and nurses), who, considering the limitations of the outpatient context (logistics, waiting spaces, etc.), could have provided a vaccination every ten minutes, equivalent to a rate of 672 total inoculations per day.

In 2022, a reduction in anti-SARS-CoV-2 vaccine administrations (about 72,000 doses compared with 300,000 in 2021) made it possible to increase the use of CVPs for ordinary vaccinations. In addition to anti-TBE vaccinations, administered in large numbers both in the adult (13,898 doses) and pediatric (1788 doses) population, vaccination campaigns were aimed at the developmental-age and adult population (herpes zoster and pneumococcus). Furthermore, in the period March–May 2022, CVPs made it possible to manage the operations deriving from the international crisis in Ukraine by issuing STPs (temporary health cards for temporarily present foreigners) and administering anti-SARS-CoV-2 vaccinations—both ordinary and booster.

In 2023, with the resumption of activity at the ordinary clinics and in consideration of the small number of anti-SARS-CoV-2 vaccinations administered, unlike previous years, the ULSS 1 Dolomiti managed the anti-TBE vaccination campaign (9181 doses administered as of 30 June 2023), the vaccination campaigns of the adult population (Herpes zoster and pneumococcus) and the catch-up of adolescents. The vaccination sessions organized in the first half of 2023 recorded 19,843 administrations, of which 13,040 were in the adult population group and 6803 in the pediatric group.

Below is Table 3, displaying the number of doses administered in active drive-in and building CVPs, divided by year and age group.

Table 4 and Table 5 show the analysis of the critical issues that emerged in the territory of the ULSS 1 Dolomiti, with the related solutions adopted and the good practices that ultimately emerged.

## 4. Discussion

The anti-SARS-CoV-2 vaccination campaign required an enormous organizational effort on the part of all health authorities, entrusting governance and coordination to the prevention department [15]. The experience gained represents a fundamental pillar for possible future pandemics, helping to guarantee preparedness and readiness should it become necessary to organize a new population vaccination campaign. This knowledge has additional relevance in light of the provisions of the new Veneto regional pandemic plan (PANFLU) [16].

With the possibility of vaccinating different age groups in all stages, the progress of the anti-SARS-CoV-2 vaccination campaign has increased the requests for vaccination doses in a fluctuating manner, a condition imposed by the variable availability of doses over time. All of this has led to a need for vaccination that is not always programmable. The need to guarantee vaccination coverage as extensively and rapidly as possible has stimulated a search for vaccination solutions, such as CVPs in buildings and drive-in sites. In some phases of the vaccination campaign, in order to promote an increase in coverage for certain segments of the population, the possibility of free access has been endorsed. This circumstance potentially involved high population numbers, and as such it was necessary to provide vaccines and staff capable of dealing with large numbers of users. In this case, the optimal solution proved to be CVPs.

Comparing the traditional outpatient model with the CVP building and/or drive-in modality, with the same number of HCWs involved, our study shows that the innovative solution that was adopted is capable of guaranteeing delivery of triple the number of vaccinations in a single day. On the other hand, it is necessary to consider the need for support from personnel (external and otherwise) responsible for logistics and management of waiting spaces and pre- and post-vaccination surveillance.

The drive-in method made it possible to reach far more people and facilitate access to the peripheral areas, even for those who had difficulties walking, such as the elderly and other fragile categories of people. The use of their own vehicle also made it easier for these people to undress (comfortable and practical clothing was already requested in the invitation); the help of family members then made it possible to reduce waiting times to a minimum, with a policy of staying in one’s car limiting challenges associated with travel and any sudden changes in temperature. In fact, during the post-vaccination wait period, during which the patient remained within a defined area with a limited perimeter, checks were carried out by HCWs and volunteers in a constant and effective manner.

Overall, from the experience of the extraordinary anti-SARS-CoV-2 vaccination campaign first and foremost, and, secondly, from the other vaccinations necessarily administered via the CVPs during the pandemic, prevention departments had the unique opportunity to govern an unprecedented and continually evolving moment in history, one whose unpredictable problems led to the development of unprecedented strategies. In other words, the reprogramming, dictated by the pandemic, of the ordinary and extraordinary vaccination activities of prevention departments was also an opportunity to reconsider the traditional operational models, as in the analysis of the weak points and the related solutions developed in our experience, from which numerous good practices have also emerged (Table 4 and Table 5). The hope is that the latter will not be “put back in the drawer” until the next pandemic emergency, but that this moment will pave the way for their application in ordinary vaccination practice. For example, the use of innovative and effective communication channels is the subject of growing attention in the healthcare sector, and experience has now demonstrated that good communication on vaccination helps to improve vaccine acceptance by the population and contributes to increasing vaccination coverage [17,18]. This goes hand in hand with the progressive and constant computerization of the service, with a view to digital transition, through the good practices adopted in our experience of making vaccinations directly bookable by users via a dedicated online portal. However, to guarantee fairness, alongside this new practice, traditional channels have been maintained (active call via ordinary mail, phone booking, etc.) in order to reach the segments of the population that have most difficulty using digital means, especially in an area with a higher prevalence of elderly people than the regional average [19,20,21].

Finally, the involvement of private industry, both operating in the healthcare sector and otherwise, during the pandemic may have paved the way for new collaborative synergies from a “health in all policies” perspective, a cornerstone of health promotion in the new millennium [22,23].

The organizational and operational methods adopted by ULSS 1 Dolomiti are reflected in other, similar experiences described in the national and international literature. Firstly, our study is particularly in line with the results emerging from the review conducted by Gianfredi et al. at the beginning of the anti-SARS-CoV-2 vaccination campaign. This publication aimed to define organizational contours and foresee obstacles and solutions based on experience gained in previous contexts [24]. This review included 15 studies describing mathematical models, one-day field simulations or real population vaccination centers (either in large buildings or drive-ins) set up during local epidemics or influenza pandemics, e.g., the recent H1N1 outbreak. Logistical details, personnel requests and the times necessary to administer the vaccinations were subject to analysis in a fashion entirely analogous with our experience. With the net of known heterogeneity between the different studies considered, the review revealed that each individual required between 14 and 30 min from acceptance to administration of the vaccine. In terms of total vaccinations, hundreds were administered per hour and thousands per day in both the drive-in and building settings, with evident variability depending on the enlisted personnel.

A similar organizational blueprint also emerged from Trainor’s description of the anti-SARS-CoV-2 campaign in a local area in the United States, in which vaccinations took place in cinemas, hospitals, and religious institutes, and where the drive-in mode was reserved for the elderly and, in general, for individuals with walking difficulties, with contributions from external bodies in terms of both logistics and personnel, as occurred in our area [25].

We found only one, English language, analysis, conducted through interviews with subjects variously involved in the administration of anti-SARS-CoV-2 vaccines, that highlighted the critical issues and strengths of the different vaccination settings and which adhered to the organization of our study. The emphasis on location was relevant as in some situations the methods perceived as best are those that allow for a more “familiar” and “local” approach with users (specifically, primary care and pharmacies), while CVPs are perceived as being at greater risk of creating unequal access in the population (difficult accessibility for those who live further away, difficulty in booking the vaccine online for the elderly, etc.) [26]. This study, however, also recognizes the CVPs’ unique ability to cope with large-scale vaccination requests, as well as their greater organizational potential in the storage and correct conservation of available vaccines and in the possibility of implementing recovery actions through active boosters.

Furthermore, the vaccination organization set up in the Belluno province during the anti-SARS-CoV-2 campaign complied with the dictates laid out the Italian Society of Hygiene, Preventive Medicine and Public Health in terms of the traditional building and drive-in models. Additionally, the measures taken corresponded with the checklist presented by Capolongo et al. regarding the criteria for validating traditional building-type CVPs and with the good practices identified in a recent Canadian publication that sought to identify a vaccination path using systematic logic. This latter study paid reference to the building model, but was easily applicable to the drive-in model [27,28,29].

## 5. Conclusions

The anti-SARS-CoV-2 vaccination campaign placed prevention departments in charge of an unprecedented challenge. From this crisis, innovative management methodologies have emerged. Many of these had been occasionally tested during local epidemics or in anticipation of influenza pandemics with a more limited impact but were fully implemented through the active involvement of HCWs with different educational and operational backgrounds working in synergy [30]. In the mission to vaccinate the entire world population as quickly as possible, critical issues were resolved and required best practices emerged in a way that had never before been seen in the history of public health. By comparing literature data at a global level, we developed mass vaccination models according to a tailor-made logic, adhering to territorial and population peculiarities. In this context, in ULSS 1 Dolomiti, a health authority comprising an almost exclusively mountainous area, vaccination provision was initiated via drive-in, with very satisfactory results, both from the point of view of acceptance by users and in organizational terms.

Finally, considering the dictates of the PANFLU, knowledge gained from the COVID-19 experience, including the rapid establishment of mass vaccination centers capable of promptly vaccinating large volumes of the population and the implementation of good communication and organizational practices, must remain easily usable in the future. This will be necessary in relation to the organization of any ordinary administration and recovery campaigns, as well as in emergency contexts, in order to guarantee timely and effective public health responses.

## Figures and Tables

**Table 1:** Territorial vaccination clinics of the ULSS 1 Dolomiti, resident and domiciled population belonging to each territorial clinic and number of weekly hours dedicated to vaccination activity per clinic.

Clinic	Population Age Group <16 Years	Population Age Group ≥16 Years	Total Population	Vaccination Clinic Time (Hours/Week)
Agordo	1786	16,640	18,426	12
Alpago	939	8371	9310	3.5
Auronzo	537	5680	6217	1.5
Belluno	5305	44,643	49,948	25.5
Cortina d’Ampezzo	872	8355	9227	3
Feltre	4349	38,141	42,490	12.5
Longarone	699	7630	8329	6
Mel	1463	12,048	13,511	3
Pieve di Cadore	930	9434	10,364	4
S. Stefano di Cadore	658	6075	6733	2
Sedico	2878	22,628	25,506	6

**Table 2:** CVPs opened in ULSS1 Dolomiti during the COVID-19 pandemic, with related features.

Location and Type of CVPs	n. Maximum Daily Vaccinations	n. Operators/ n. Vaccine Lines	Strengths	Areas of Improvement	Ready Availability of the Structure	Separate Routes	Note
CVP “Palaskating Sedico”	1200 (8 working hours); on average 30 users/h per vaccination line	10 operators/5 vaccination lines, 3 operators for vaccine preparation, 2 operators for reception, 1 responsible doctor	- Ample parking - Ease of access - Heated room	- External waiting area not covered - Non-air-conditioned room	No	No	Active from May 2021 to June 2022
CVP “Comelico”			- Easily accessible by the entire population - Heated room	- External waiting area not covered	Yes	No	Active on request
CVP “Centro prelievi” Feltre	360 (4 working hours); on average 30 users/h per vaccination line	6 operators/3 vaccination lines, 3 operators for vaccine preparation, 2 operators for reception, 1 responsible doctor	- CVP in a hospital context - Easily accessible by the entire population - Heated room	- External waiting area not covered	Yes	No	
CVP “prefabbricato lato sud—Belluno hospital”	120 (4 working hours); on average 30 users/h per vaccination line	2 operators/1 vaccination line, 2 operators for vaccine preparation, 1 reception operator, 1 responsible doctor	- CVP in a hospital context - Heated room	- Narrow spaces - Possibility of use only in the afternoon or on Sundays - External waiting area not covered	Yes	No	Active on request
CVP “Salce”	480 (4 working hours, possibility of activation up to 8 h); on average 30 users/h per vaccination line	8 operators/4 vaccination lines, 3 operators for vaccine preparation, 2 operators for reception, 1 responsible doctor	- Easily accessible by the entire population - Covered external waiting area	- Limited parking	Yes	No	Active from October 2022
Drive-in “San Gervasio—Belluno Hospital”	360 (4 working hours); on average 30 users/h per vaccination line	6 operators/3 vaccination lines, 3 operators for vaccine preparation, 1 reception operator, 1 for post vaccination control, 1 operators per registration, 1 responsible doctor	- Drive-in in a hospital context - Easily accessible by the entire population	- Drive-in in hospital contexts with difficulties for ambulances to travel if queues arise - In order not to obstruct hospital traffic, use is only possible in the afternoon and on Saturdays and Sundays - Difficulties in post-vaccination checks and possible rescue operations - Difficulties for operators related to external work	No	No	Active until September 2022
Drive-in “Paludi”	360 (4 working hours); on average 30 users/h per vaccination line	6 operators/3 vaccination lines, 3 operators for vaccine preparation, 1 reception operator, 1 for post vaccination control, 1 operators per registration, 1 responsible doctor	- No obstruction to ordinary traffic	- Difficulty in access by the population due to the distance from the town centre - Distance from the hospital, therefore need for an ambulance on site - Difficulties in post-vaccination checks and possible rescue operations - Difficulties for operators linked to a workplace located in external area	Yes	No	Active until October 2021
Drive-in “Feltre”	360 (4 working hours); on average 30 users/h per vaccination line	6 operators/3 vaccination lines, 3 operators for vaccine preparation, 1 reception operator, 1 for post vaccination control, 1 operators per registration, 1 responsible doctor	- Easily accessible by the entire population	- Difficulties in post-vaccination checks and possible rescue operations - Difficulties for operators linked to a workplace located in external area	Yes	Yes	Active until December 2021
Drive-in “Agordo”	180 (3 working hours); on average 30 users/h per vaccination line	4 operators/2 vaccination lines, 2 operators for vaccine preparation, 1 reception operator, 1 for post vaccination control, 2 operators per registration, 1 responsible doctor	- Easily accessible by the entire population	- Difficulties in post-vaccination checks and possible rescue operations - Difficulties for operators linked to a workplace located in external area	Yes	Yes	
Drive-in “Cadore”	240 (4 working hours); on average 30 users/h per vaccination line	4 operators/2 vaccination lines, 2 operators for vaccine preparation, 1 reception operator, 1 for post vaccination control, 2 operators per registration, 1 responsible doctor	- Easily accessible by the entire population	- Difficulties in post-vaccination checks and possible rescue operations - Small post-vaccination waiting car park - Possible obstruction to ordinary traffic - Difficulties for operators linked to a workplace located in external area	Yes	Yes	
Drive-in “Cortina ice rink”	240 (4 working hours); on average 30 users/h per vaccination line	4 operators/2 vaccination lines, 2 operators for vaccine preparation, 1 reception operator, 1 for post vaccination control, 2 operators per registration, 1 responsible doctor	- Easily accessible by the entire population	- Difficulties in post-vaccination checks and possible rescue operations - Small post-vaccination waiting car park - Difficulties for operators linked to a workplace located in external area	Yes	No	Active on request

**Table 3:** Data on volumes of vaccination administrations (anti-SARS-CoV-2 and all other non-COVID-19 vaccinations) in CVP buildings/drive-ins, divided by age group and year, from 2020 to the first half of 2023. Source: Veneto Region Portal QLIK VIEW.

	2020			2021			2022			2023 I Semester		
	Adults	Pediatrics	Total	Adults	Pediatrics	Total	Adults	Pediatrics	Total	Adults	Pediatrics	Total
Drive-in “Agordo”	1900	162	2062	24,709	853	25,562	7167	513	7680	769	23	792
Drive-in “Cadore”	3827	496	4323	36,627	1221	37,848	11,250	866	12,116	1625	211	1836
CVP “Feltre”	7393	849	8242	67,391	3847	71,238	16,739	4079	20,818	3435	1837	5272
Drive-in “Belluno Hospital”	12,144	1217	13,361	37,822	1479	39,301	8772	578	9350	-	-	-
CVP “Cortina ice rink”	-	-	-	2417	133	2550	1027	85	1112	-	-	-
CVP “Palaskating Sedico”	-	-	-	79,910	9888	89,798	39,388	10,968	50,356	-	-	-
Drive-in “Paludi”	-	-	-	39,303	997	40,300	154	10	164	-	-	-
CVP “Salce”	-	-	-	-	-	-	5806	1356	7162	7211	4732	11,943
TOTAL	25,264	2724	27,988	288,179	18,418	306,597	90,303	18,455	108,758	13,040	6803	19,843

**Table 4:** Analysis of the critical issues and business solutions that emerged in ULSS 1 Dolomiti during the anti-COVID-19 vaccination campaign.

Organizational Critical Issues	Organizational Solution Adopted
Initial difficulty in obtaining vaccine doses in relation to demand.	Centralization of the preparation of doses at the hospital pharmacy to optimize the number of doses prepared.
Centralized preparation of vaccines and distribution of doses within 6 h of preparation.	Involvement of civil protection to deliver doses and avoid waste.
Lack of adequate premises for mass vaccination.	Use of the drive-in method already established for testing (swabs and serological tests for COVID-19).
Vast and mountainous territory.	Organization of stable drive-ins in four locations (Agordo, Tai di Cadore, Belluno, Feltre) in conjunction with other forms of response in the area, including the involvement of GPs, also in local sessions.
Difficulty in reaching expected coverage standards.	Repeated invitations, active calls, involvement of positive reinforcements (for example young athletes), call centers for doubters, active calls by GPs to their doubtful clients.
Variability of the vaccination calendar (number of doses to be administered, availability and indications for use of the vaccine).	Centralized management of agendas and dedicated vaccination sessions.
Difficulty in booking online (strong presence of elderly people in the area, not accustomed to using computers).	Call-center, paper invitation delivered by post.
Insufficiency of personnel to carry out the entire vaccination campaign.	Involvement of hospital HCWs on a voluntary basis in an objective project, stipulation of voluntary contracts with self-employed doctors and nurses, including retired ones.
Unfavorable weather and climate conditions.	Use of heating solutions in drive-ins and provision of adequate outdoor clothing.

**Table 5:** Best practices that emerged in ULSS 1 Dolomiti in carrying out and organizing the anti-SARS-CoV-2 vaccination campaign.

Best Practices Adopted
Best practice 1: Involve private industry in the site identification and management of the main CVP.
Best practice 2: Carry out vaccinations at the Belluno Hospital clinics for people at risk of adverse reactions.
Best practice 3: Identify a call center, active every day to provide information to users. These should be trained on the basis of the progression of the pandemic event (swabs, vaccines, vaccination doses, contraindications, and EU-digital COVID certificate).
Best practice 4: Creation of a web platform for online bookings, with the possibility of controlling appointment diaries, depending on the available vaccine supplies.
Best practice 5: Constantly updated use of social media and involvement of the world of sport or people in activites such as positive reinforcement of the vaccination campaign.

## Data Availability

Not applicable.
